# Raising cigarette excise tax to reduce consumption in low-and middle-income countries of the Asia-Pacific region:a simulation of the anticipated health and taxation revenues impacts

**DOI:** 10.1186/s12889-018-6096-z

**Published:** 2018-10-19

**Authors:** Li-Ming Ho, Christian Schafferer, Jie-Min Lee, Chun-Yuan Yeh, Chi-Jung Hsieh

**Affiliations:** 1 Department of Marine Leisure Management, National Kaohsiung University of Science and Technology, Kaohsiung, Taiwan; 2 Department of International Trade, Overseas Chinese University, Taichung, Taiwan; 3 Department of Shipping and Transportation Management, National Kaohsiung University of Science and Technology, 142, Hai-Chuan Rd. Nan-Tzu, Kaohsiung, Taiwan; 4 Department of Finance, National Changhua University of Education, Changhua, Taiwan

**Keywords:** Smoking, Taxation, Smoking-attributable mortality, Low and middle income countries, Asia Pacific

## Abstract

**Background:**

According to the World Health Organization (WHO), 80% of the world’s smokers live in low- and middle-income countries. Moreover, more than half of the world’s smoking-addicted population resides in the Asia-Pacific region. The reduction of tobacco consumption has thus become one of the major social policies in the region. This study investigates the effects of price increases on cigarette consumption, tobacco tax revenues and reduction in smoking-caused mortality in 22 low-income as well as middle-income countries in the Asia-Pacific region.

**Methods:**

Using panel data from the 1999–2015 Euromonitor International, the World Bank and the World Health Organization, we applied fixed effects regression models of panel data to estimate the elasticity of cigarette prices and to simulate the effect of price fluctuations.

**Results:**

Cigarette price elasticity was the highest for countries with a per capita Gross National Income (GNI) above US$6000 (China and Malaysia), and considerably higher for other economies in the region. The administered simulation shows that with an average annual cigarette price increase of 9.51%, the average annual cigarette consumption would decrease by 3.56%, and the average annual tobacco tax revenue would increase by 16.20%. The number of averted smoking-attributable deaths (SADs) would be the highest in China, followed by Indonesia and India. In total, over 17.96 million lives could be saved by tax increases.

**Conclusion:**

Excise tax increases have a significant effect on the reduction of smoking prevalence and the number of averted smoking-attributable deaths. Middle- and upper-middle income countries would be most affected by high-taxation policies.

## Background

### Smoking and tobacco taxes

An estimated 680 million smokers, more than half of the world’s smoking-addicted population, resides in the Asia-Pacific region (incl. Western Pacific and Southeast Asia) [1, 2]. Studies by the World Health Organization (WHO) suggest that 80% of the world’s smokers live in low-income and middle-income countries and that more than 80% of those dying from lung cancer by 2030 will be from those countries [3]. Low and middle-income countries, including those in the Asia-Pacific region, are comparatively more affected by cigarette addiction than other countries.

In 2012, the Asian Development Bank estimated that, in the absence of intervention, smoking would eventually kill about 267 million current and future cigarette smokers in China, India, the Philippines, Thailand, and Vietnam. The study concluded that tax-induced price increases would effectively reduce the number of smokers and incidences of smoking-related deaths, while generating substantial additional tobacco tax revenues [4].

In most Asia-Pacific countries, tobacco pricing is subject to specific excise taxes, whereas ad valorem taxes are less common. In an increasing number of cases, specific excise as well as ad valorem taxes (mixed excise) are levied (Table 1). A specific excise tax is levied on quantity, whereas an ad valorem excise tax is levied as a percentage of the value of tobacco products. Although mixed excise taxation is considered the most effective in reducing nicotine consumption, the predominant taxation regime varies by region and is mostly determined by tobacco industry characteristics, public awareness and socio-political considerations [5]. In recent years, mixed excise taxation has been the preferred form of taxation in Europe and has gained popularity in Africa and Southeast Asia, while countries in the Western Pacific maintain a preference for specific excise tax only [6].

**Table 1:** Cigarette excise taxes structure and cigarette consumption, and retail prices from 2006 to 2015 in 22 Asia- Pacific countries

Countries	Excise tax structure /level				Per capita legal cigarette consumption of population aged over 15 (No. packs)			Real retail price of a pack of legal cigarettes (US$)			Age-standardized adult smoking prevalence (%)
	Type of excise tax applied	Specific excise		Ad valorem excise	2006	2015	Change (2006~ 2015)	2006	2015	Change (2006~ 2015)	
		% of retail price	LCU (per stick)	% of retail price							
low and lower middle income country											
GNI≦US$999											
Cambodia	Ad valorem excise	0	–	13.15	18.53	22.47	21.20%	1.34	1.34	−0.20%	18
Nepal	Specific excise	16.29		0	3.02	2.7	−10.60%	0.8	1.09	36.00%	27.1
Bangladesh	Ad valorem excise	0	–	61	19.46	23.02	18.30%	0.46	0.5	8.90%	22.3
Myanmar	Ad valorem excise	0	–	50	8.35	7.86	−5.90%	0.24	0.12	−48.30%	23.8
US$1000≦GNI≦US$1999 US$											
India	Mixed excise	42.25	0.9~ 2.3	1.27	4.52	3.9	−13.70%	1.28	1.62	26.90%	13.4
Laos	Mixed excise	6.25	–	1.4	23.67	27.38	15.70%	0.66	0.68	3.20%	35.3
Vietnam	Ad valorem excise	0	–	32.5	44.54	47.97	7.70%	0.82	0.58	−29.70%	18.6
Bhutan	–	–	–	–	6.55	5.52	−15.70%	0.73	1.18	62.00%	28.8
Papua New Guinea	Specific excise	26.42	–	0	37.72	25.37	−32.70%	0.93	0.99	6.00%	26.7
Solomon Islands	Specific excise	19.15	–	0	1.09	0.64	−41.50%	2.89	3.67	26.80%	19.28
US$2000≦GNI≦US$2999											
Indonesia	Mixed excise	40.91	150~ 380	4.09	33.32	50.52	51.60%	1.17	1.34	14.20%	31
Mongolia	Specific excise	33.26	–	0	24.43	36.65	50.00%	0.83	0.78	−6.10%	24.3
Sri Lanka	Mixed excise	59.15	–	3.91	13.83	11.84	−14.40%	1.49	2.07	39.50%	12.8
Vanuatu	Specific excise	44.44		0	2.32	1.71	−26.50%	3.5	3.53	0.60%	26
US$3000≦GNI≦US$3999											
Philippines	Specific excise	63.55	0.6~ 1.25	0	48.62	42.12	−13.40%	0.79	1.18	49.80%	20.1
Samoa	Specific excise	42.32		0	1.67	1.21	−27.60%	3.79	3.53	−6.60%	37.3
Tonga	Specific excise	58.82	–	0	2.5	1.83	−26.90%	2.89	3.27	13.10%	35.9
upper middle countries											
US$4000≦GNI≦US$5999											
Fiji	Specific excise	31.05	–	0	27.34	19.8	−27.60%	2.16	2.61	21.10%	10.9
Maldives	No excise	0	–	0	8.89	7.57	−14.90%	0.78	1.16	48.10%	24
Thailand	Mixed excise	2.86	–	63.72	30.78	35.92	16.70%	1.34	1.83	36.50%	16.1
GNI > US$6000											
China	Mixed excise	0.6	0.003	29.3	74.52	95.94	28.70%	1.04	2.59	150.40%	30.5
Malaysia	Mixed excise	41.67	0.22	8.93	32.51	21.14	−35.00%	2.33	2.97	27.40%	23

Global tobacco consumption and taxation policies have been strongly influenced by the WHO Framework Convention on Tobacco Control (WHO FCTC). The framework went into effect in 2005 and aims at the reduction of global tobacco use through price (tax) and non-price related measures as well as efforts to curb illicit tobacco trade. Mechanisms for scientific and technical cooperation and exchange of information assist participating parties to address tobacco control issues more effectively than previous global WHO tobacco control initiatives [6]. Since the signing of the WHO framework convention (FCTC), low- and middle-income countries in the Asia-Pacific region have substantially increased tobacco taxes. Nevertheless, tobacco taxation has remained below the WHO FCTC declared best-practice tax rate of over 75% of retail price (Table 1) [7].

### Price elasticities and cigarette demand

Cigarette price elasticity in low-income or middle-income countries usually ranges between − 0.5 to − 1.05 [8–11]. However, elasticity is reportedly much lower (− 0.25 to − 0.5) in high-income countries, where cigarette affordability tends to be comparatively higher [8, 12, 13]. As for Asia-Pacific countries, estimates of cigarette price elasticity are rarely reported since research has so far focused on a restricted number of countries. That is, most studies have been cross-sectional and time series analyses on China, Bangladesh, India, Indonesia, Myanmar, Nepal, Sri Lanka, and Thailand, report price elasticity figures for China between − 0.01 to − 0.84 [14–22], for Bangladesh between − 0.26 to − 0.49 [23–25], for India between 0.15 to − 1.021 [26–28], for Indonesia at − 0.62 [29], for Myanmar at − 0.34 [28], for Nepal at − 0.88 [10], for Sri Lanka between − 0.29 to − 0.68 [30], and for Thailand between − 0.09 to − 0.67 [31, 32].

### The goals of this study

The main contribution of this study is to estimate the price elasticity of cigarette demand in low-income and middle-income countries of the Asia-Pacific region applying panel data analysis. If compared with time-series or cross-sectional studies, panel data analyses allow researchers to control for unobservables that threaten causal inference in observational studies and offer more opportunities to explore patterns of causal relationships over longer time spans [33, 34]. Furthermore, potential effects of excise tax increases on cigarette consumption, tax revenues, and the death toll of smoking are analysed by administering hypothetical price increases based on figures derived from maximum and mean increments in cigarette prices during 1999–2015. The findings of this study may serve as an important reference for health management authorities in Asia-Pacific countries to revise tobacco prevention and control policies.

## Methods

### Study design and data

In this study, data of 22 Asia-Pacific countries were collected to construct a cigarette demand structure model. Kiribati, North Korea, Nauru, Timor-Leste, and the Marshall Islands were excluded from the study because of lack of data. One dependent variable and three independent variables were considered. Per capita cigarette consumption for those aged 15 and over was chosen as the dependent variable. Independent variables comprised cigarette prices, gross national income (GNI) per capita, and current status of WHO FCTC ratification.

Data regarding cigarette consumption and prices were extracted from the 1999–2015 Euromonitor International market research database [35]. Euromonitor International is recognized as a leading independent provider of global business intelligence, specializing in creating worldwide data and analysis on consumer products and services. Consumption of cigarette products was calculated based on annual cigarette consumption per capita for those aged 15 and over. The retail price for a pack of cigarettes in each country was calculated by dividing cigarette sales revenues by cigarette consumption, which was further deflated using consumer price indexes [36].

The GNI per capita data were accessed from the World Bank’s database, converted to US dollars using the World Bank Atlas method [36], divided by the midyear population, and deflated based on consumer price indexes. The current status of WHO FCTC ratification provides information on whether individual countries have ratified the convention. Countries that have already ratified the convention were given a 1 value. Data regarding the ratification of the WHO FCTC were obtained from the 2015 WHO report on global tobacco epidemic [7].

### Data characteristics

Table 1 lists all variables used in the analysis and visualizes their characteristics. The structural composition of excise taxes on tobacco products in the observed countries is also shown in Table 1. Total excise taxes comprise specific excise, ad valorem excise, or mixed excise. Specific excise is the most common form. According to the 2015 WHO report on global prevalence of tobacco products, the specific excise tax proportion of retail price in 2014 was the highest in the Philippines (63.55%), followed by Sri Lanka (59.15%) and Tonga (58.82%), whereas specific excise taxes accounted for less than 50% in the remaining countries. High levels of ad valorem taxes were levied on tobacco products in Thailand (63.72), Bangladesh (61%), Myanmar (50%), Vietnam (32.5%), and China (29.3%). Total taxes were the highest in Thailand (66.58), followed by the Philippines (63.55%), Sri Lanka (63.06) and Bangladesh (61%). The Maldives did not levy taxes on cigarettes. Low total tax rates were also in evidence in Laos (7.65%), Cambodia (13.15%), and Nepal (16.29%).

In 2015, per capita cigarette consumption in the 22 Asia-Pacific countries for those aged 15 years and over was the highest in China at 95.94 packs, followed by Indonesia (50.52 packs), Vietnam (47.97 packs), and the Philippines (42.12 packs), while consumption in the other Asia-Pacific countries was below 40 packs (Table 1). With the exceptions of Indonesia, Mongolia, China, and Cambodia where cigarette consumption showed a rising trend, Indonesia experienced the highest growth (51.6%), followed by Mongolia (50%) and China (28.7%). Consumption in other countries assumed a downward trend with the Solomon Islands exhibiting the greatest decrease (41.5%), followed by Malaysia (35%).

According to WHO estimates, the smoking prevalence rate of populations aged 15 or older in the 22 observed low-income and middle-income countries is below 40%. It is the highest in Samoa at 37.3%, followed by Tonga (35.9%), Laos (35.3%), China (30.5%), Bhutan (28.8%), and Nepal (27.1%). India (13.4%), Sri Lanka (12.8%), and Fiji (10.9%) exhibit the lowest prevalence rates.

As shown in Table 1, in 2015, the average real retail price for a pack of cigarettes was the highest in the Solomon Islands at US$3.67 per pack, followed by Vanuatu (US$3.53). Considering the fluctuation of real retail prices of cigarettes between 1999 and 2015, cigarette prices across the Asia-Pacific region generally showed a rising trend, with China experiencing the greatest increase (150.4%), followed by Bhutan (62%), the Philippines (49.8%) and the Maldives (48.1%), while cigarette prices in Myanmar and Vietnam decreased by 48.3%, and 29.7%, respectively (Table 1).

### Empirical specification and analysis

To calculate cigarette price elasticity, a cigarette demand structure model was constructed using cigarette consumption as the dependent variable and cigarette price, gross national income (GNI), and the ratification of WHO FCTC as explanatory variables.

To estimate price elasticities of demand for cigarettes, we have applied a conventional demand model with a linear equation in this study. We assume

1 $$\ln {C}_{it}={\beta}_{1i}+{\beta}_2\ln {P}_{it}+{\beta}_3\ln {GNI}_{it}+{\beta}_4{FCTC}_{it}+{\varepsilon}_{it}$$

where C_it_ is the annual cigarette consumption per capita in the population aged 15 and older in country i in year t (1999–2015), β_1i_ is the intercept for country i, P_it_ is the cigarette real retail price per pack of 20 cigarettes in country i in year t, GNI_it_ is per capita gross national income in country i in year t, and FCTC_it_ is a dummy variable to describe the state of ratification of the WHO FCTC in country i in year t.

Endogeneity must be considered for the regression analysis to avoid biased estimates. Among the regressors used in our analysis, cigarette price has been reported as the most likely sources of possible endogeneity in studies on cigarette consumption [37, 38]. We addressed this issue by using cigarette price and consumer price indexes in periods t–1 as instruments for cigarette price. A weak identification test (Kleibergen-Paap Wald F) was carried out to verify instrument relevance [39].

A Hausman test was applied to determine which model should be used for the equation estimation. A rejection of the test is taken to mean that the key random-effects assumption is false and in such cases the fixed-effects estimates should be used [40].

To determine the effects of cigarette price increases on cigarette consumption, cigarette consumption in 2015 was set as the baseline for this study. Maximum and mean increments in cigarette prices during 1999–2015 were applied to simulate changes in future cigarette consumption based on the cigarette price elasticity estimated in this study. Changes in tobacco tax revenues were calculated based on changes in consumption due to price increases. Percentages of price increases were calculated using the yearly mean and maximum price increases between 1999 and 2015.

Previous research has shown that cigarette price elasticity is likely to be affected by income levels [20, 41]. To account for this income threshold effect, we performed our analysis using clusters of countries with different income levels. That is, the observed 22 countries were first grouped into two clusters according to World Bank classification: (a) low- and lower-middle income countries as well as (b) upper-middle income countries. The latter group of countries was then divided into two clusters and the first into four. Gross national income per capita data for the year 2015 (Atlas method) were used in the clustering and consideration was given to obtain clusters of approximately equal size and significantly different GNI values (Table 1).

The number of averted smoking-attributable deaths (SADs) derived from the simulated impact of price increments on the reduction in smokers and was adjusted for the fact that smoking cessation still carries considerable risks of early death [42]. The applied mortality adjustment factors were calculated for each country surveyed, assuming that 95%, 75%, 70%, 50% and 10% of those who ceased smoking when aged 15 to 29, 30 to 39, 40 to 49, 50 to 59 and at least 60 years, respectively, would remain unaffected by their previous smoking habits. Data on population stratification were extracted from the World Bank database [36].

## Results

### Regression results

As price elasticities may change as a result of changes in income levels, we performed six panel regressions to investigate the effect of cigarette prices on cigarette consumption. Results of the administered Hausman test t showed that the models were statistically significant at the 5% level for all six samples, indicating that the fixed effects model should be administered for these samples (Table 2). We thus applied the fixed effects model for all six samples.

**Table 2:** Results of fixed effect regression models of panel data, 22 Asia- Pacific countries (1999–2015)

Dependent variable: (lnCit)						
Countries	Low- and lower-middle income				Upper-middle-income	
Independent variables	Group1 (GNIit≦US$999)^a^	Group 2 (US$1000≦GNIit≦US$1999)^b^	Group 3 (US$2000≦ GNI_it_≦US$2999)^c^	Group 4 (US$3000≦ GNIit≦US$3999)^d^	Group 5 (US$4000≦GNIit≦US$5999)^e^	Group 6 (GNIit > US$6000)^f^
Constant	1.435 (0.203)**	0.919 (0.182)**	−0.072 (0.256)	0.831 (0.564)	0.269 (0.341)	−0.757 (0.552)
ln P_it_	−0.037 (0.068)	− 0.146 (0.089)	− 0.488 (0.289)**	− 0.267 (0.238)	− 0.614 (0.155)**	−1.304 (0.306)**
lnGNI_it_	− 0.161 (0.076)**	0.046 (0.061)	0.408 (0.078)**	0.017 (0.165)	0.327 (0.098)**	0.769 (0.161)**
FCTC_it_	0.02 (0.012)	−0.048 (0.005)**	−0.154 (0.027)**	− 0.072 (0.023)**	−0.045 (0.023)**	− 0.103 (0.046)**
Observations	68	102	68	51	51	34
Number of country	4	6	4	3	3	2
Hausman test	60.07**	7.41	34.56**	34.65**	39.08**	20.81**
R^2^	0.256	0.533	0.919	0.849	0.657	0.387
Wald F test	91.131**	146.45**	23.555**	23.663**	79.333**	21.077**

Ln: natural logarithm; C_it_: the annual cigarette consumption per capita in the population aged 15 and older in country i in year t; P_it_: the cigarette real retail price per pack of 20 cigarettes in country i in year t; GNI_it_: per capita gross national income in country i in year t; FCTC_it_: the dummy variable to describe the state of ratification of the WHO Framework Convention on Tobacco Control in country i in year t; ** denote statistically significant at 5%; Standard errors are shown in parentheses. Sargan test stands for overidentification test of all instruments. Wald F test stands for weak identification test (Kleibergen-Paap rk F statistic)

^a^Group1 countries (GNI per capita of US$999 or less): Cambodia, Nepal, Bangladesh, Myanmar

^b^Group 2 countries (GNI per capita between US$1000 and US$1999): India, Laos, Vietnam, Bhutan, Papua New Guinea, Solomon Islands

^c^Group 3 countries (GNI per capita between US$2000 and US$2999): Indonesia, Mongolia, Sri Lanka, Vanuatu

^d^Group 4 countries (GNI per capita between US$3000 and US$3999): Philippines, Samoa, Tonga

^e^Group 5 countries (GNI per capita between US$4000 and US$5999): Fiji, Maldives, Thailand

^f^Group 6 countries (GNI per capita of US$5999 or more): China, Malaysia

Instrument relevance (Kleibergen-Paap Wald F) was tested for the instrumental variables used in the regression to determine whether the instruments are invalid. The results for the lagged (t-1) cigarette price and consumer price indexes as the instrumental variables showed that the instrument is not weak for the majority of income clusters (see Table 2). Based on the results, we decided to use the fixed effects model with cigarette price as the instrument variable in our six panel regressions. Moreover, fixed effects estimation was paired with cluster-robust variance estimation as to account for heteroskedasticity and un-modeled dependence among the errors [43].

### Elasticity estimates

A conventional regression model was used in this study to estimate the elasticity of demand for cigarettes. The cigarette consumption, cigarette price, and income variables were all logarithmically transformed to estimate elasticity. There were differences in the cigarette price elasticity of each income group in the Asia-Pacific region (Table 2). When GNI per capita was higher than US$6000 (Group 6), cigarette price elasticity was the highest at − 1.304 and income elasticity 0.769. When GNI per capita was between US$4000 and US$5999 (Group 5), cigarette price elasticity reached − 0.614 and income elasticity, 0.327. Cigarette price elasticity for countries with a GNI per capita lower than US$1000 was at − 0.037, the lowest value among the six income groups, and income elasticity at − 0.161. In addition, previous ratification of the WHO FCTC showed a negative and statistically significant impact on cigarette consumption.

### The effects of cigarette prices on cigarette consumption, tobacco tax revenue, and smoking-related deaths

To determine the effects of tax-induced cigarette price increases on cigarette consumption, cigarette prices and consumption levels of 2015 were set as the baseline for this study. The maximum and mean annual increments in cigarette prices during 1999–2015 were used to simulate changes in future cigarette consumption based on price elasticity estimated in this study. In both scenarios, increases in cigarette prices (mean and maximum) reduced cigarette consumption the most in China (price mean and max: 9.45% and 50.96%; consumption mean and max: 12.32% and 66.45%), followed by Malaysia (price mean and max: 7.68% and 17.04%; consumption mean and max: 10.01% and 22.22%). The other countries with a large reduction in cigarette consumption were the Maldives (mean: 5.53%, max: 21.3%), Sri Lanka (mean: 4.95%, max: 9.76%), and Indonesia (mean: 4.72%, max: 13.06%). The simulation result also suggests that with an average annual cigarette price increase of 9.51% during the observed period of 1999–2015, the average annual cigarette consumption would decrease by 3.56% in the 22 Asia-Pacific countries (Table 3).

**Table 3:** Impact of real retail cigarette price increases in 22 Asia- Pacific countries between 1999 and 2015 on cigarette consumption per capita, tax revenue, reduction in no. of smokers and reduction in smoking-attributable deaths

Countries	Annual max and mean increase % in real retail cigarette price		Annual max and mean decrease % in per capita cigarette consumption		Annual max and mean increase % in cigarette tax revenue		Reduction in no. of smokers due to cigarette price increase		Max and mean reduction in SADs	
	Max (%)	Mean (%)	Max (%)	Mean (%)	Max (%)	Mean (%)	Max	Mean	Max	Mean
	Low and lower middle income									
GNI≦US$999										
Cambodia	26.52	6.46	−0.98	−0.24	198.71	48.77	−19,105	− 4654	− 4983	− 1214
Nepal	25.86	10.71	−0.96	− 0.40	156.27	65.09	−47,965	− 19,865	− 12,974	− 5373
Bangladesh	17.11	8.72	−0.63	−0.32	27.24	13.93	−160,793	−81,947	−46,839	−23,871
Myanmar	88.48	20.05	−3.27	−0.74	167.89	39.06	− 316,592	−71,741	−93,553	−21,200
US$1000≦GNI≦US$1999										
India	23.28	11.74	−3.40	−1.71	48.03	24.67	−4,028,113	−2,031,359	−1,213,670	−612,049
Laos	21.01	8.85	−3.07	−1.29	262.11	112.45	−49,408	−20,812	−12,382	− 5216
Vietnam	28.55	6.34	−4.17	−0.93	80.02	18.40	−544,700	−120,960	− 166,079	−36,881
Bhutan	27.15	10.17	−3.96	−1.48	–	–	− 6202	− 2323	− 1738	− 651
Papua New Guinea	34.56	12.73	−5.05	−1.86	119.16	45.43	−62,714	−23,100	−16,569	− 6103
Solomon Islands	39.13	14.46	−5.71	−2.11	186.95	71.80	− 3915	− 1447	− 1001	− 370
US$2000≦GNI≦US$2999										
Indonesia	26.77	9.68	−13.06	−4.72	38.65	15.77	−7,242,053	−2,618,718	−2,303,697	−833,014
Mongolia	31.28	8.65	−15.26	−4.22	66.90	21.46	−78,062	−21,587	−21,569	− 5964
Sri Lanka	19.99	10.14	−9.76	−4.95	18.85	10.33	−199,680	−101,288	−71,206	−36,119
Vanuatu	15.38	7.02	−7.51	−3.43	24.51	11.83	− 1921	− 877	− 500	− 228
US$3000≦GNI≦US$3999										
Philippines	30.18	7.22	−8.06	−1.93	35.61	9.21	−1,088,283	− 260,351	− 294,598	− 70,477
Samoa	17.29	8.20	−4.62	−2.19	34.35	16.76	− 2048	− 972	− 584	− 277
Tonga	39.64	10.13	−10.58	−2.70	49.68	14.05	− 2558	−654	− 747	− 191
	Upper middle income									
US$4000≦GNI≦US$5999										
Fiji	24.99	6.58	−15.34	−4.04	52.79	16.30	−10,603	− 2792	− 3342	− 880
Maldives	34.69	9.01	−21.30	−5.53	–	–	−13,551	− 3520	− 3366	− 874
Thailand	12.44	5.18	−7.64	−3.18	9.62	4.35	− 693,909	− 288,943	−256,608	−106,851
GNI > US$6000										
China	50.96	9.45	−66.45	−12.32	−9.27	15.39	−232,702,235	−43,152,200	−86,472,151	−16,035,358
Malaysia	17.04	7.68	−22.22	−10.01	3.97	3.64	−1,152,312	− 519,352	− 357,217	− 160,999
All 22 Asia Pacific region	29.65	9.51	−11.09	−3.56	63.58	16.20	−248,426,711	−49,349,461	−91,355,371	−17,964,159

SADs: smoking-attributable death. The number of SADs averted was calculated according to Goodchild et al. [14]: Reduction in SADs = Reduction in no. of smokers multiplied by the corresponding mortality adjustment factor

Tobacco tax revenue in 2015 was used as the baseline to predict future effects of mean changes in cigarette prices on tobacco tax revenue (Table 3). The simulation result shows that the average annual tobacco tax revenue would increase by 16.2%. Laos had the highest percentage increase in tobacco tax revenue (mean and max increase: 112.45% and 262.11%), followed by Solomon Islands (mean and max increase: 65.09% and 156.27%), Nepal (mean and max increase: 71.8% and 186.95%) and Cambodia (mean and max increase: 48.77% and 198.71%).

Results of the simulation also showed that excise tax increases could potentially avert a total of 17.96 million smoking-related deaths in low and middle-income countries of the Asia-Pacific region. Specifically, about 16 million deaths could be avoided in China; 833,014 in Indonesia; 612,049 in India; 160,999 in Malaysia; 106,851 in Thailand; and 70,477 in the Philippines (Table 3).

## Discussion

Previous studies have emphasized the effectiveness of tax-induced price increases to curb tobacco consumption and their subsequent positive impact on public health and finances [44]. Notwithstanding, regional socio-economic and cultural variations may produce divergent results. This study found that among low-income as well as in middle-income countries in the Asia Pacific region, countries with a per capita GNI above US$6000 exhibited the highest cigarette price elasticity. That is, most low- and middle-income countries in the region showed considerably lower elasticity figures, contradicting previous research findings, suggesting that price elasticity in less developed countries would be higher than in advanced economies [44]. Industrial counter measures as well as public policy deficiencies may explain the differences in price elasticity estimates. That is, the tobacco industry reportedly adopts a low-price strategy to mitigate tax-induced effects on cigarette prices [45]. For example, in countries such as Bangladesh, Myanmar and Vietnam real retail prices of cigarettes haven fallen over the years despite higher tobacco taxes (Table 1). Moreover, previous studies have highlighted the necessity of implementing other anti-smoking measures outlined in the WHO FCTC, such as cessation programs, bans on advertisements, and improving public awareness, in combination with higher tobacco taxation measures [7, 13]. According to WHO reports, however, non-tax related tobacco control measures are generally inadequately implemented in less developed countries, thus reducing the effects of excise taxation policies [6].

According to our simulation, cigarette consumption would decrease the most in China, where low cigarette prices have contributed to high levels of smoking prevalence (30%) with consumption amounting to one-third of global cigarette demand [7]. Among the observed Asia-Pacific countries, Indonesia, the Maldives, Sri Lanka, Mongolia and Malaysia would also be significantly affected by additional excise taxation policies. Specifically, excise taxation would not only reduce cigarette consumption, but would also lead to additional tax revenues, which could be utilized to enforce existing anti-smoking policies as well as to finance future policy instruments. Currently, a total of 10 out of the 22 countries observed in the study have taxation levels below 40%, while no taxes are levied on tobacco in the Maldives. That is, tax rates are far below the best-practice taxation rate of over 75% suggested by the World Health Organisation (WHO). Moreover, economic growth in low- and middle-income countries has made tobacco affordable to increasing numbers of consumers. The proportion of excise taxes on cigarette retail prices would thus have to be increased accordingly to compensate for increases in purchasing power.

In general, higher tobacco taxation in countries with low (or no) cigarette taxes and high smoking prevalence reportedly has a large impact on cigarette consumption. Less developed economies levying ad valorem excise on tobacco, such as Cambodia, Bangladesh, Myanmar and Vietnam, could however introduce (additional) specific excise taxes to obtain a greater impact on cigarette prices and to mitigate the impact tobacco companies may have in attempts to counteract ad valorem taxes. Currently, smoking prevalence in these countries may exhibit limited negative effects on public health and finances, but restricted state capacities to accumulate necessary funds to implement anti-smoking measures are likely to constitute considerable burdens on national health care systems in the future. Adopting specific excise taxation on tobacco as well as establishing externally-funded anti-smoking agencies may be beneficial to alleviate the negative effects of smoking on public health.

In our study, a statistically significant relationship between the ratification of the WHO FCTC and subsequent reductions in cigarette consumption could be established for all the six income clusters, except for the income group with the lowest GNI per capita (Cambodia, Nepal, Bangladesh, and Myanmar). Moreover, previous research on cigarette price elasticity and taxation effects in the Asia-Pacific applied cross-sectional methods to investigate individual countries, whereas transnational data were analysed in this study. Integrated analysis of transnational data may, however, lead to incorrect inferences, because different countries have different cigarette consumption structures. Public health authorities in the Asia-Pacific region are thus advised to establish cigarette consumption databases and market-monitoring mechanisms to facilitate long-range tracking and analysis. Moreover, demographic factors and other effects of tax-induced price increases, such as brand switching, consumption of inferior tobacco products, and illicit trade as well as their impact on consumption were not addressed in this study and should be discussed in future research.

## Conclusion

This study estimated that price elasticities of each income group in the Asia-Pacific region ranged from − 0.037 to − 1.304. Countries with a per capita GNI above US$6000 (China and Malaysia) exhibited the highest cigarette price elasticity. That is, most low income as well as middle-income countries in the region showed considerably lower elasticity figures during the study period. Higher elasticity is likely to be obtained by introducing/increasing specific excise taxes and concerted efforts to implement non-price (tax) related WHO FCTC measures, such as cessation programs, bans on advertisement, and improving public awareness. The subsequent increase in tobacco tax revenues would also be instrumental in covering expenditures related to such tobacco prevention and control programs.

## Abbreviations

FCTC: Framework Convention on Tobacco Control
GNI: Gross National Income
SADs: Smoking-Attributable Deaths
WHO: World Health Organization

## References

[1] World Health Organization (1999). The world health report 1999: making a difference.

[2] World Health Organization (2013). The tobacco free initiative in the Western Pacific region.

[3] Hammond SK, London ED, Pogun S (2009). Global patterns of nicotine and tobacco consumption. Nicotine Psychopharmacology (Handbook of Experimental Pharmacology).

[4] Asian Development Bank: Tobacco taxes: a win-win measure for fiscal space and health. Philippines. Asian Development Bank 2012. Available from: https://www.adb.org/sites/default/files/publication/30046/tobacco-taxes-health-matters.pdf [Accessed 6 June 2017].

[5] World Health Organization: Tobacco Tax Levels and Structure: A Theoretical and Empirical Overview. WHO Technical Manual on Tobacco Tax Administration 2010, 28–53. Geneva: World Health Organization. Available from: http://www.who.int/tobacco/publications/economics/tax_administration/en/ [Accessed 1 Aug 2018].

[6] World Health Organization: Global Progress Report 2016. Geneva: World Health Organization. Available from: http://www.who.int/fctc/reporting/2016_global_progress_report.pdf [Accessed 1 Aug 2018].

[7] World Health Organization (2016). WHO report on the global tobacco epidemic, 2015.

[8] Gallet CA, List JA (2003). Cigarette demand: a meta-analysis of elasticities. Health Econ.

[9] Hidayat B, Thabrany H (2010). Cigarette smoking in Indonesia: examination of a myopic model of addictive behaviour. Int J Environ Res Public Health.

[10] Karki YB, Pant KD, Pande BR: A study on the economics of tobacco in Nepal. HNP discussion paper series. Economics of tobacco control 2003, paper no. 13. Washington: The World Bank http://documents.worldbank.org/curated/en/320681468323404408/pdf/288870Karki1A0Study1whole.pdf

[11] Mushtaq N, Mushtaq S, Beebe LA (2011). Economics of tobacco control in Pakistan: estimating elasticities of cigarette demand. Tob Control.

[12] Chaloupka FJ, Hu TW, Warner KE, Jacobs R, Yurekli A, Jha P, Chaloupka FJ (2000). The taxation of tobacco products. Tobacco control in developing countries.

[13] Chaloupka FJ, Straif K, Leon ME (2011). Effectiveness of tax and price policies in tobacco control. Tob Control.

[14] Goodchild M, Perucic AM, Nargis N (2016). Modelling the impact of raising tobacco taxes on public health and finance. Bull World Health Organ.

[15] Hu T-w, Mao Z, Shi J, Chen W (2010). The role of taxation in tobacco control and its potential economic impact in China. Tob Control.

[16] Hu T-w (2002). Effects of cigarette tax on cigarette consumption and the Chinese economy. Tob Control.

[17] Kostova D, Chaloupka FJ, Yurekli A, Ross H, Cherukupalli R, Andes L, Asma S (2014). A cross-country study of cigarette prices and affordability: evidence from the global adult tobacco survey. Tob Control.

[18] Mao ZX, Yang GH, Ma H (2003). Adults’ demand for cigarettes and its determinants in China. Soft Science of Health.

[19] Mao ZZ, Hu T-w, Yang GH (2005). New estimate of the demand for cigarettes in China. Chin J Health Econ.

[20] Mao ZZ, Hu T-w, Yang GH (2005). Price elasticities and impact of tobacco tax among various income groups. Chin J Evid Based Med.

[21] Mao ZZ, Jiang JL (1997). Demand for cigarette and pricing policy. Chin Health Econ.

[22] Mao ZZ, Jiang JL (1997). Determinants of the demand for cigarettes: a cross-sectional study. Chin Health Serv Manage.

[23] Barkat A, Chowdhury AU, Nargis N, Rahman M, Khan S, Kumar A (2012). The economics of tobacco and tobacco taxation in Bangladesh.

[24] Nargis N, Ruthbah UH, Fong GT (2010). Taxation of Tobacco Products in Bangladesh: Findings from the 2009 ITC Bangladesh Survey.

[25] Nargis N, Ruthbah UH, AKMG H, Ashiquzzaman SM (2011). Pricing and Taxation of Tobacco Products in Bangladesh: Findings from Wave 1 (2009) and Wave 2 (2010) of the ITC Bangladesh Survey.

[26] Guindon GE, Nandi A, Chaloupka FJ, Jha P: Socioeconomic differences in the impact of smoking tobacco and alcohol prices on smoking in India. NBER working paper No. 17580. Cambridge : National Bureau of Economic Research 2011. http://www.nber.org/papers/w17580.pdf

[27] John RM (2008). Price elasticity estimates for tobacco products in India. Health Policy Planning.

[28] Selvaraj S, Srivastava S, Karan A (2015). Price elasticity of tobacco products among economic classes in India, 2011-2012. BMJ Open.

[29] Adioetomo SM, Djutaharta T, Hendratno (2005). Cigarette consumption, taxation and household income: Indonesia case study. HNP discussion paper.

[30] Arunatilake N. An economic analysis of tobacco demand in Sri Lanka. Sri Lanka Econ J. 2002;3(1):96–120.

[31] Sarntisarta I, Supakankunti S, Teerachaisakul M, Chuensukkasemkul K, Kaluntakaphanc N: The economics of tobacco in Thailand. HNP Discussion Paper, Economics of Tobacco Control 2003, Paper no 15. Washington DC: World Bank. Available from: http://siteresources.worldbank.org/HEALTHNUTRITIONANDPOPULATION/Resources/281627-1095698140167/Sarntisart-AnEconomic_Thailand_whole.pdf [Accessed 6 June 2017].

[32] Supakorn B: Demand analysis of aggregate cigarette consumption in Thailand, 1976–81 [mimeograph]. Bangkok: Health Systems Research Institute; 1993.

[33] Halaby CN (2004). Panel models in sociological research: theory into practice. Annu Rev Sociol.

[34] Hsiao C (2014). Analysis of panel data (3rd ed.).

[35] Euromonitor International (database online) (2014). Tobacco: global. Passport database. Euromonitor.

[36] World Bank (2014). World development indicators (WDI).

[37] Ngo A, Cheng KW, Chaloupka FJ, Shang C (2017). The effect of MPOWER scores on cigarette smoking prevalence and consumption. Prev Med.

[38] Wilkins N, Yurekli A, Hu T: Economic Analysis of Tobacco Demand. Tool 3: Demand Analysis. In: Yurekli A, Beyer J (eds.) Economics of Tobacco Toolkit. World Bank 2007, Washington 1–96. http://siteresources.worldbank.org/INTPH/Resources/3Demand.pdf

[39] Finlay K, Magnusson LM, Schaffer ME: WEAKIV: Stata module to perform weak-instrument-robust tests and confidence intervals for instrumental-variable (IV) estimation of linear, probit and tobit models. 2017. Available at: http://ideas.repec.org/c/boc/bocode/s457684.html

[40] Wooldridge JM. Introductory economics: a modern approach (4th ed). Mason, Ohio: South-Western Cengage Learning; 2009.

[41] Huang BN, Yang CW (2006). Demand for cigarettes revisited: an application of the threshold regression model. Agric Econ.

[42] Ranson MK, Jha P, Chaloupka FJ, Nguyen SN, Jha P, Chaloupka FJ (2000). The effectiveness and cost-effectiveness of price and other tobacco control policies. Tobacco control in developing countries.

[43] Bertrand M, Duflo E, Mullainathan S (2012). How much should we trust differences-in-differences estimates?. Q J Econ.

[44] International Agency for Research on Cancer: Effectiveness of tax and price policies for tobacco control. IARC handbooks of cancer prevention: tobacco control 2011, 14. Lyon: International Agency for Research on Cancer. Available from: http://publications.iarc.fr/_publications/media/download/4018/05229a5e57f58b0bf51364dd0f3329d45c898839.pdf [Accessed 06 June 2017].

[45] Doku D. The tobacco industry tactics – a challenge for tobacco control in low and middle income countries. Afr Health Sci. 2010;10(2):201–3. http://publications.iarc.fr/_publications/media/download/4018/05229a5e57f58b0bf51364dd0f3329d45c898839.pdf. PMC2956281 21326977

